# Correction to: Investigation of a Suspect Severe Acute Respiratory Syndrome Coronavirus-2 and Influenza A Mixed Outbreak: Lessons Learned for Long-Term Care Facilities Nationwide

**DOI:** 10.1093/cid/ciac264

**Published:** 2022-05-24

In the original publication of this article [Schrodt CA, Malenfant JH, Hunter JC et al. Investigation of a Suspect Severe Acute Respiratory Syndrome Coronavirus-2 and Influenza A Mixed Outbreak: Lessons Learned for Long-Term Care Facilities Nationwide. Clin Infect Dis; S77-S80, Volume 73 Issue Supplement_1, July 15, 2021], the product OSOM Ultra Flu A&B Test was incorrectly referred to as OSOM Ultra Plus Flu A&B Test. This has been corrected in the version currently available online.

